# Choroidal Structure in Children with Anisohypermetropic Amblyopia Determined by Binarization of Optical Coherence Tomographic Images

**DOI:** 10.1371/journal.pone.0164672

**Published:** 2016-10-13

**Authors:** Tomo Nishi, Tetsuo Ueda, Yuutaro Mizusawa, Kayo Shinomiya, Kentaro Semba, Yoshinori Mitamura, Shozo Sonoda, Eisuke Uchino, Taiji Sakamoto, Nahoko Ogata

**Affiliations:** 1 Department of Ophthalmology, Nara Medical University, Kashihara, Nara, Japan; 2 Department of Ophthalmology, Institute of Biomedical Sciences, Tokushima University Graduate School, Kuramoto, Tokushima, Japan; 3 Department of Ophthalmology, Kagoshima University Graduate School of Medical and Dental Sciences, Sakuragaoka, Kagoshima, Japan; Massachusetts Eye & Ear Infirmary, Harvard Medical School, UNITED STATES

## Abstract

**Purpose:**

To compare the choroidal structure of the subfoveal area in the eyes of children with anisohypermetropic amblyopia to that of the fellow eyes and to age-matched controls using a binarization method of the images obtained by enhanced depth imaging optical coherence tomography (EDI-OCT).

**Methods:**

This study was performed at Nara Medical University Hospital, Tokushima University Hospital, and Kagoshima University Hospital, Japan. Forty amblyopic eyes with anisohypermetropic amblyopia and their fellow eyes (5.9 ± 2.1 years, mean ± standard deviation), and 103 age-matched controls (6.7 ± 2.4 years) were studied. The control eyes were divided into myopic, emmetropic, and hyperopic eyes. The total choroidal area, luminal area and stromal area of the subfoveal choroid were measured by the binarization method. The luminal/stromal ratio and the axial length of the amblyopic eyes were compared to that of the control eyes.

**Results:**

The total choroidal area in the amblyopic eyes was significantly larger than that of the fellow eyes (*P* = 0.005). The luminal/stromal ratio was significantly larger in the amblyopic eyes than that of the fellow eyes (*P*<0.001) and the control hyperopic eyes (*P*<0.001). There was a significant negative correlation between the luminal/stromal ratio and the axial length in the control eyes (*r* = -0.30, *P* = 0.001), but no significant correlation was found in the amblyopic eyes.

**Conclusions:**

The choroidal structure of the amblyopic eyes was different from that of the fellow and the control hyperopic eyes. The choroidal changes are related to amblyopia.

## Introduction

The choroid plays an important role in not only providing nutrients to the inner parts of the eye but also in modulating the refractive state of the eye.[1] There have been studies which reported that the choroidal thickness (CT) in healthy eyes decreases with increasing age[2][3]. Thus, the choroidal thickness of healthy children is thicker than that of healthy adults.[4][5] In addition, the choroid of amblyopic eyes is thicker than that of healthy children.[6][7] Several studies have reported on the volume and structure of the choroid in healthy adult eyes[8][9] and healthy children eyes,[10] however the structural changes of the choroidal of amblyopic children eyes have not been determined.

Sonoda et al recently reported on a new method to analyze the choroidal structure quantatively and objectively.[11][12] They used an open access software called ImageJ and were able to differentiate the luminal and stromal areas of the choroid and quantify the size of these areas objectively. This method has been termed the binarization method, and it has not been used in eyes with anisohypermetropic amblyopia.

Thus, the purpose of this study was to compare the choroidal structure of children with anisohypermetropic amblyopia to that of age-matched controls. The choroidal structure was determined by the binarization method of the optical coherence tomographic (OCT) images obtained by enhanced depth imaging OCT (EDI-OCT). We investigated whether the luminal and stromal areas of the choroid of amblyopic eyes were different from that of fellow and normal control eyes.

## Patients and Methods

This was a retrospective, cross sectional observational study conducted at the Nara Medical University, Tokushima University, and Kagoshima University Hospitals from April 2012 to May 2016. The protocol of this study conformed to the tenets of the Declaration of Helsinki and was approved by Internal Review Boards (IRBs) of the Nara Medical University Hospital, Tokushima University Hospital, and Kagoshima University Hospital. The approvals were obtained before any of the measurements were made. After informing all of the patients and parents on the purpose of this study, an informed consent was obtained to perform the original measurements and to use the information gathered in future research studies. An informed consent was obtained by written from their fathers or mothers on behalf of the children enrolled in our study.

Forty eyes of 40 patients with anisohypermetropic amblyopia and their fellow eyes were studied. The mean age of the patients was 5.9 ± 2.1 years (± standard deviation) with a range of 3 to 11 years. An eye was classified as being amblyopic when the best-corrected visual acuity (BCVA) was worse than 20/30 in one eye and was at least two Snellen lines worse than that of the fellow eye. Anisometropia was defined as being present when the difference in the refractive error between the two eyes was greater than 2 diopters.

In addition, 103 right eyes of 103 age-matched controls (6.7±2.4 years) were studied in the same way. The control group was composed of children whose BCVA was ≥20/20, whose age was from 3 to 12 years, and who had no ocular disorders in either eye. The control group was divided into three groups according to their refractive errors (spherical equivalent); a myopic group with refractive error ≦-0.5 diopter (D), an emmetropic group with refractive error between -0.5 D to +0.5 D, and hyperopic group with refractive error ≧ +0.5 D. The same examination procedures were used on the amblyopic eyes and the right eyes of the controls. The axial length and visual acuity of 22 of the amblyopic and fellow eyes of the amblyopic patients and 20 of the eyes of the control patients have been reported.[6] All of the participants had eye examinations including slit-lamp biomicroscopy, extraocular motility assessments, subjective cycloplegic refractions (1% cyclopentlate and 2.5% phenylephrine), dilated funduscopy, and SD-OCT recordings. Patients with organic eye diseases, history of intraocular surgery, laser treatment, cataract, glaucoma, premature infants, or any retinal disorders were excluded.

The visual acuity was measured with a standard Snellen chart, and the decimal visual acuity was converted to the logarithm of the minimal angle of resolution (logMAR) units for the statistical analyses. The refractive error was measured with an autorefractometer RC-5000 (TOMEY, Nagoya, Japan) and KR8100, RM8900 (Topcon, Tokyo, Japan). The axial length of the eye was measured with the A-mode ultrasound IOL Master (Carl Zeiss Meditec, Dublin, CA), AL-2000 (TOMEY, Nagoya, Japan), and OA-1000 (TOMEY, Nagoya, Japan).

### Enhanced depth imaging optical coherence tomography (EDI-OCT)

The choroidal area was measured on the images obtained by the Heidelberg Spectralis spectral-domain optical coherence tomographic instrument (Heidelberg Engineering, Heidelberg, Germany; SD-OCT) with the EDI program from all patients and controls. All examinations were performed between 11:00 to 15:00 hours to avoid diurnal variations in the choroidal thickness.[13][14] All images were recorded by an experienced ophthalmologist or by one of the authors. Two observers who were masked on whether the eye was amblyopic selected the best image and independently measured the choroidal area. The final area was calculated as the arithmetic mean of the values obtained by the two observers. The inter-observer reproducibility was evaluated by intraclass correlation coefficients (ICCs).

### Evaluation of choroidal area by binarization method of EDI-OCT images

The choroidal area was binarized by an open access software, ImageJ (version 1.50a: provided in the public domain by NIH, Bethesda, Maryland, USA) according to the method described in detail by Sonoda et al.[11] Briefly, the examined area was selected to be 1500 μm wide with the margins 750 μm nasal and 750 μm temporal to the fovea. This area was bordered vertically by the RPE and the chorioscleral border. The choroidal area was set with the ImageJ ROI Manager. Three choroidal vessels with lumens larger than 100 lumen were randomly selected by the Oval Selection Tool on the ImageJ tool bar and the average reflectivity of these areas was determined. The average brightness was set as the minimum value of lumens to minimize the noise in the OCT images. Then the image was converted to 8 bits and adjusted by the Niblack Auto Local Threshold method. The binarized image was converted to red, green, blue image again, and the luminal area was determined using the Threshold Tool. After adding the data of the distance between the pixels, the choroidal area, luminal area, and stromal area were automatically calculated. The light pixels were defined as the stromal area and the dark pixels as the luminal area.

### Statistical analyses

The data are expressed as the means ± standard deviations (SDs). The age, BCVA, axial length, and refractive error (spherical equivalent) of the amblyopic, fellow eyes were compared to the control eyes by one-way ANOVA. The choroidal area between amblyopic eyes and fellow eyes were compared between-groups by one-way analysis of covariance (ANCOVA) in which the differences in the visual acuity, refractive error, and axial length were left out. The significance of the differences in the choroidal area between the control hyperopic, emmetropic, and myopic eyes was determined by ANCOVA in which the differences in the age, sex distribution, visual acuity, refractive error, and axial length were left out. The differences in the choroidal area between the amblyopic and control hyperopic eyes were determined by ANCOVA which controlled for the differences of the visual acuity, refractive error, and axial length. If a significant difference was found by ANCOVA, pairwise comparison was performed with the Bonferroni test. The correlation between the choroidal area and axial length was determined for all of the patients and controls by Pearson`s correlation coefficient. A *P* <0.05 was taken to be significant. Statistical analysis was performed using licensed statistical software (SPSS version 21.0; SPSS Inc., Chicago, IL).

## Results

### Demographic data

There was no significant difference in the ages between the amblyopic group and control group (Table 1). The mean BCVA was 0.40 ± 0.21 logMAR units in the amblyopic eyes, -0.04 ± 0.08 logMAR units in the fellow eyes and -0.19 ± 0.34 logMAR units in the control eyes. The mean BCVA was significantly worse in the amblyopic eyes than that of the fellow and the control eyes (*P* <0.001, ANOVA, Table 1).

**Table 1:** Demographic information of the amblyopic patients and controls.

	Amblyopic eyes	Fellow eyes	Control eyes	*P* value^1^
	(n = 40)	(n = 40)	(n = 103)	
**Age (year)**	5.9 ± 2.1	5.9 ± 2.1	6.7 ± 2.4	0.079
**Male/Female**	16 / 24	16 / 24	38 / 65	0.893
**Visual acuity (logMAR)**	0.40 ± 0.21	-0.04 ± 0.08	-0.19 ± 0.34	<0.001
**Spherical Equivalent (D)**	4.57 ± 1.55	1.35 ± 1.45	0.19 ± 2.25	<0.001
**Axial Length (mm)**	21.2± 0.6	22.1 ± 0.7	22.7 ± 1.3	<0.001

^1^ ANOVA.

Data are expressed as means ± standard deviations.

The mean refractive error was +4.57 ± 1.55 diopters (D) in the amblyopic eyes, +1.35 ± 1.45 D in the fellow eyes, and +0.19 ± 2.25 D in the control eyes (*P<*0.001, ANOVA; Table 1). The mean axial length was 21.2 ± 0.6 mm in the amblyopic eyes, 22.1 ± 0.7 mm in the fellow eyes and 22.7 ± 1.3 mm in the age-matched control eyes. The mean axial length was significantly shorter in the amblyopic eyes (*P* <0.001, ANOVA).

The inter-observer reproducibility was very high with ICC = 0.84 for the total area, 0.82 for the luminal area and 0.81 for the stromal area.

The total choroidal area in the amblyopic eyes was significantly larger than that of the fellow eyes (*P* = 0.005; Table 2). The luminal area was significantly larger in the amblyopic eyes than that of the control eyes (*P* < 0.001; Table 2). The luminal/stromal ratio was significantly larger in the amblyopic eyes than in the fellow eyes (*P* = 0.004; Table 2).

**Table 2:** Choroidal area of the amblyopic and fellow eyes.

	Amblyopic eyes	Fellow eyes	*P* value^1^
	(n = 40)	(n = 40)	
**Total Choroidal Area (μm^2^)**	564199 ± 94618	476219 ± 91802	0.005
**Luminal Choroidal Area (μm^2^)**	417454 ± 90671	323500 ± 78393	<0.001
**Stromal Choroidal Area (μm^2^)**	146745 ± 45176	152719 ± 39587	0.163

^1^ ANCOVA adjusted with axial length, spherical equivalent and visual acuity.

Data are expressed as means ± standard deviations.

The eyes in the control group were divided into hyperopic, emmetropic, and myopic eyes, and the choroidal parameters were compared to the corresponding parameters in the amblyopic eyes. There were no significant differences in the total, luminal, and stromal area of the choroid among the eyes in the control groups (Table 3).

**Table 3:** Comparison of the choroidal area between the control hyperopic, emmetropic, and myopic eyes.

	Control eyes			*P* value^1^
	Hyperopic eyes	Emmetropic eyes	Myopic eyes	
	(n = 40) (≧0.5D)	(n = 32)	(n = 31) (≦-0.5D)	
**Total area (μm^2^)**	564971 ± 110664	538630 ± 117419	469700 ± 124104	0.806
**Luminal area (μm^2^)**	375954 ± 74564	358422 ± 75590	300226 ± 85947	0.668
**Stromal area (μm^2^)**	189017 ± 48344	180208 ± 48121	169473 ± 43155	0.959
**Luminal / stromal ratio**	2.1 ± 0.6	2.0 ± 0.4	1.8 ± 0.3	0.670

^1^ANCOVA adjusted with age, sex, axial length, and visual acuity.

Data are expressed as means ± standard deviations.

The amblyopic eyes were compared to control hyperopic eyes. There was no significant difference in the total area between amblyopic (564199 ± 94618 μm^2^) and control hyperopic eyes (564971 ± 110664 μm^2^; *P* = 0.785, ANCOVA adjusted for visual acuity, refractive error, and axial length; Table 4). The luminal area was significantly larger in amblyopic eyes (417454 ± 90671 μm^2^) than that in control hyperopic eyes (375954 ± 74564 μm^2^) (*P* = 0.041, ANCOVA adjusted for visual acuity, refractive error, and axial length; Table 4). The stromal area was significantly smaller in amblyopic eyes (146745 ± 45176 μm^2^) than that in control hyperopic eyes (189017 ± 48344 μm^2^) (*P* = 0.003, ANCOVA adjusted for visual acuity, refractive error, and axial length; Table 4). The luminal/stromal ratio was significantly larger in the amblyopic eyes than in the control eyes (*P* <0.001, ANCOVA adjusted for visual acuity, refractive error, and axial length, Table 4).

**Table 4:** Difference between amblyopic eyes and control hyperopic eyes.

	*P* value^1^
**Total Area**	0.785
**Luminal area**	0.041
**Stromal area**	0.003
**Luminal / stromal ratio**	<0.001

^1^ANCOVA adjusted with axial length, spherical equivalent and visual acuity.

### Representative amblyopic eye and control eye

**Fig 1:**
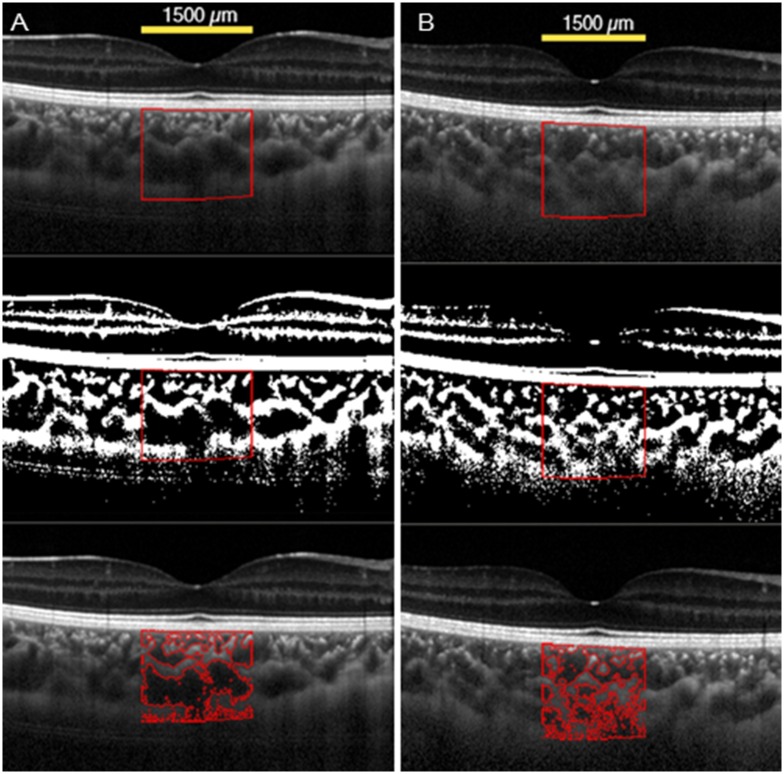
Enhanced depth spectral-domain optical coherence tomographic (EDI-OCT) images of the choroid of an amblyopic eye and a control eye. (A) Representative image of the amblyopic eye. The amblyopic eye has large luminal area. (B) Representative image of the control hyperopic eye. The control hyperopic eye has small luminal area. The choroidal area measured was 1500 μm wide with the margins 750 μm nasal and 750 μm temporal to the fovea. Vertically, the area extended from the retinal pigment epithelium to the chorioscleral border (yellow line). The measured area of the choroid is demarcated (Top). The image is converted to a binary image by the Niblack method of ImageJ (Middle). The dark area which is luminal area is traced by the red line (Bottom).

The findings in an 8-year-old amblyopic patient are shown in Fig 1A. Our initial examination showed that his BCVA was 0.2 logMAR units in the right eye and 0 logMAR units in the left eye. Slit-lamp and fundus examinations showed that both eyes were completely normal. The refractive error (spherical equivalent) of the amblyopic eye was +4.75 D of the right eye. In the amblyopic eye, the total choroidal area was 625,356 μm^2^, and luminal/stromal ratio was 3.2 (Fig 1A). The choroidal structure of a 7-year-old control child is shown in Fig 1B. The visual acuity of the control eye was 0 logMAR units, and the refractive error was +4.62 D. In the control hyperopic eye, the total area was 584,278μm^2^, and the luminal/stromal ratio was 1.7 (Fig 1B). The total area and the luminal/stromal ratio of the amblyopic eye were larger than that of the control eye.

### Correlation between the luminal/stromal ratio and the axial length

**Fig 2:**
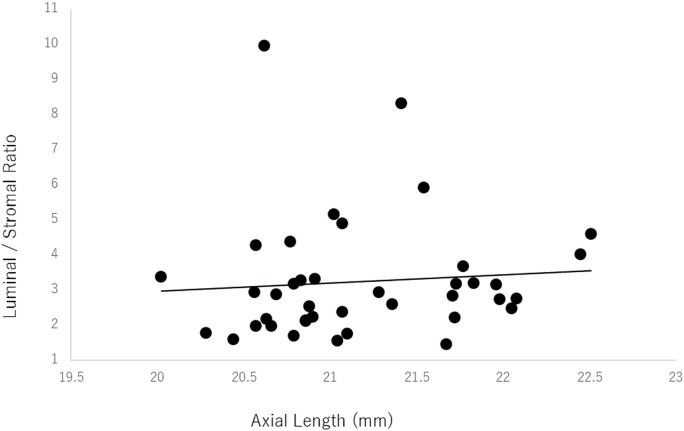
Relationship between the luminal/stromal ratio and the axial length of the amblyopic eyes. There was no significant correlation between the luminal/stromal ratio and the axial length (*r* = 0.08, *P* = 0.61 Pearson’s correlation coefficient). ●: amblyopic eyes.

**Fig 3:**
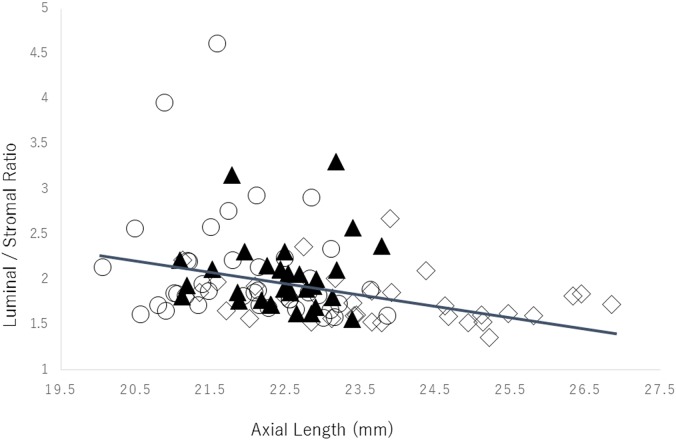
Relationship between the luminal/stromal ratio and the axial length of the control eyes. There was a weak but significant negative correlation between the luminal area of the choroid and the axial length *(r* = -0.29, *P* = 0.003 Pearson’s correlation coefficient). ○: hyperopic eyes, ▲: emmetropic eyes, ◇: myopic eyes.

In the amblyopic eyes there was no significant correlation between the luminal/stromal ratio and the axial length (*r* = 0.08, *P* = 0.61, Pearson’s correlation coefficient; Fig 2). However, a weak but significant negative correlation was found between the luminal/stromal ratio and the axial length in the control eyes (*r* = -0.29, *P* = 0.003, Pearson’s correlation coefficient; Fig 3).

## Discussion

Our analyses showed that the total choroidal area in the amblyopic eyes was significantly larger than that of the fellow eyes. The luminal area was significantly larger and the stromal area was significantly smaller in the amblyopic eyes than that in the control hyperopic eyes. Therefore, the luminal/stromal ratio of amblyopic eyes was larger than that of fellow and control hyperopic eyes. We reported earlier that the subfoveal choroidal thickness of amblyopic eyes was thicker than that of control eyes.[6] In amblyopic eyes, the choroid was the thickest in the subfoveal area followed by the temporal area,[6] while the choroid was thickest in the temporal area of the macula in the fellow and the control eyes.[6] In the present study, the total choroidal area was significantly larger in amblyopic eyes than that in the fellow eyes. This agreed with our previous report. [6]

There was a concern that the hyporeflective area in the OCT image was overestimated in the thick choroid because the signal strength was weakened by the thick outer area of the choroid. This would result in larger luminal areas. However, this is unlikely because the choroidal structure of the luminal and stromal areas in the OCT images differed significantly between amblyopic eyes and normal hyperopic eyes both of which had almost the same choroidal thickness (Table 4). Therefore, the present method is most likely valid.

Interestingly, there were no significant differences in the luminal/stromal ratio among the control myopic, emmetropic, and hyperopic eyes. In the control eyes, the luminal/stromal ratio was significantly associated with the axial length, but in the amblyopic eyes, the luminal/stromal ratio was not significantly associated with the axial length. The differences in the refractive errors did not affect the choroidal area and constitution in the three normal control groups. Thus, the difference of the choroidal constitution between anisohypermetropic amblyopic eyes and normal control eyes would most likely be associated with the amblyopia.

The choroidal structure of normal children has not been examined in detail. Ramrattan et al performed histological studies of eyes of children, and they reported that aging caused a decrease in the density and the diameter of the choriocapillaries.[15] It is generally believed that children have larger choroidal luminal area and thicker choroid than adults.[15] Barteselli et al reported that adults men had a larger choroidal volume compared to that of women,[16] however in children there is no consensus about sex differences. In adults with anisometropic amblyopia, there was no significant difference in the subfoveal choroidal thickness between amblyopic and fellow or control eyes. [17,18] In children, the choroid may have greater flexibility compared to that in adults. We found that amblyopic eyes had larger luminal areas than that of normal children. The larger luminal area in amblyopic eyes may be due to the immaturity of the eyes.

The enlargement of the luminal area in amblyopic eyes may be associated with the shorter axial length.[15][19] However, the smaller stromal area is unique to the amblyopic eyes, and we suggest that this is because the nonvascular smooth muscle cells exist in the stromal area and are most concentrated in the temporal quadrant of the posterior pole.[20] A choroidal thinning during accommodation is the greatest in the temporal and inferotemporal choroid in humans which corresponds to the distribution of the nonvascular smooth muscle cells.[21] It is known that the amblyopic eyes have lower accommodative amplitudes.[22] Because the intrinsic choroidal neurons are found in close contact with the contractile nonvascular smooth muscle cells and receive a copy of the signal sent to the ciliary body during accommodation, it has been hypothesized that the nonvascular smooth muscle cells are involved in the modulation of the choroidal thickness to stabilize the foveal position during accommodation.[23]

Taking all of these observations together,[6][20–23] we assume that the nonvascular smooth muscle cells in the stromal area of the choroid may be different in number, distribution, and function in amblyopic eyes from that in normal children,. A fewer number and/or different distribution of the nonvascular smooth muscle cells of amblyopic eyes can result in a smaller stromal area and also may induce less accommodation and result in a thicker choroid. Troilo et al[24] hypothesized that a thicker choroid slows the growth of the eye during normal development in primates either by being a barrier to the diffusion of growth factors or as a mechanical buffer to limit the elongation of the eye. Thus, we suppose that the larger luminal area inhibits the eye growth and induces hyperopia in accordance with our previous reports. [6]

The strength of this study is that the structure of the choroid was measured quantitatively. The measurements of the hyporeflective and hyperreflective areas were done automatically so that the results were essentially objective. This method required only a commercially available OCT instrument with the publically available ImageJ software. In addition, the intraclass agreement of this method was high as reported earlier. [11,12]

Our study has limitations including the small number of the subjects. Additional studies including a larger number of subjects will be necessary. Future longitudinal studies of the subjects will determine the changes of the choroidal structure of amblyopic eyes and control eyes. These follow-up studies should disclose whether improvements of the visual acuity in amblyopic eyes are reflected in changes of the choroidal structure.

Our findings are important because they highlight the fact that choroidal changes in the eye are related to amblyopia. The choroidal structure of amblyopic eyes was different from that of normal control hyperopic eyes with the luminal areas of amblyopic eyes being significantly larger than that of control hyperopic eyes.
