# Correction: Choroidal Structure in Children with Anisohypermetropic Amblyopia Determined by Binarization of Optical Coherence Tomographic Images

**DOI:** 10.1371/journal.pone.0168826

**Published:** 2016-12-15

**Authors:** Tomo Nishi, Tetsuo Ueda, Yuutaro Mizusawa, Kayo Shinomiya, Kentaro Semba, Yoshinori Mitamura, Shozo Sonoda, Eisuke Uchino, Taiji Sakamoto, Nahoko Ogata

There is an error in Table 2. The row with values for “Luminal/Stromal Ratio” is missing. Please see the correct Table 2 here.

**Table 2:** Choroidal area of the amblyopic and fellow eyes.

	Amblyopic eyes	Fellow eyes	*P* value^1^
	(n = 40)	(n = 40)	
**Total Choroidal Area (μm^2^)**	564199 ± 94618	476219 ± 91802	0.005
**Luminal Choroidal Area (μm^2^)**	417454 ± 90671	323500 ± 78393	<0.001
**Stromal Choroidal Area (μm^2^)**	146745 ± 45176	152719 ± 39587	0.163
**Luminal/Stromal Ratio**	3.2 ± 1.7	2.3 ± 1.4	0.004

^1^ ANCOVA adjusted with axial length, spherical equivalent and visual acuity.

Data are expressed as means ± standard deviations.
